# Efficacy and cost-effectiveness of an ACT and compassion-based intervention for women with breast cancer: study protocol of two randomised controlled trials {1}

**DOI:** 10.1186/s13063-024-08626-4

**Published:** 2025-01-03

**Authors:** Inês A. Trindade, Andreia Soares, David Skvarc, Diogo Carreiras, Joana Pereira, Óscar Lourenço, Filipa Sampaio, Bruno de Sousa, Teresa C. Martins, Paula Boaventura, Joana Marta-Simões, Mind Project Team, Ana Galhardo, Ana Pereira, Bruna Veloso, Lara Palmeira, Sérgio A. Carvalho, Nuno Ferreira, Marcela Matos, Paula Castilho, Ricardo João Teixeira, Marta Viegas, Margarida Borrego, Tomás Cabral Dinis, Inês Félix Pinto, Leonor Santos Martins, Nicholas J. Hulbert-Williams, Helena Moreira

**Affiliations:** 1 Center for Health and Medical Psychology, School of Behavioural, Social and Legal Sciences, University of Örebro, Örebro, Sweden; 2 Center for Research in Neuropsychology and Cognitive and Behavioral Intervention, Faculty of Psychology and Education Sciences, University of Coimbra, Coimbra, Portugal; 3 School of Psychology, Faculty of Health, Deakin University, Geelong, VIC Australia; 4 Lusófona University, Porto, Portugal; 5 CeBER, Faculty of Economics, University of Coimbra, Coimbra, Portugal; 6 Department of Public Health and Caring Sciences, Uppsala University, Uppsala, Sweden; 7 Laboratory of Molecular Pathology, Portuguese Institute for Oncology at Coimbra Francisco Gentil, Coimbra, Portugal; 8 Centre for Neurosciences and Cell Biology, University of Coimbra, Coimbra, Portugal; 9 IPATIMUP - Institute of Molecular Pathology and Immunology of the University of Porto, Porto, Portugal; 10 i3s - Institute for Research & Innovation in Health, University of Porto, Porto, Portugal; 11 Present Address: Miguel Torga Institute (ISMT), Coimbra, Portugal

**Keywords:** Acceptance and Commitment Therapy, Breast cancer, Compassion-Focused Therapy, Mind programme, Randomised controlled trial

## Abstract

**Background:**

Breast cancer is the most diagnosed cancer in women worldwide and carries a considerable psychosocial burden. Interventions based on Acceptance and Commitment Therapy (ACT) and compassion-based approaches show promise in improving adjustment and quality of life in people with cancer. The Mind programme is an integrative ACT and compassion-based intervention tailored for women with breast cancer, which aims to prepare women for survivorship by promoting psychological flexibility and self-compassion. A pilot study of the Mind programme has shown acceptability and preliminary efficacy in improving quality of life and psychological health. This paper presents the study protocol of two randomised controlled trials that aim to test the efficacy and cost-effectiveness of an optimised version of the Mind programme in women with breast cancer.

**Methods:**

Participants will be women diagnosed with breast cancer randomly assigned to the Mind programme or a support group intervention (active control) in a 1:1 ratio for study 1, while study 2 includes one more arm (treatment as usual; inactive control) and a 2:2:1 ratio. Both interventions will be delivered weekly via an 8-session face-to-face or online group format. Data will be collected at baseline, post-treatment and 6-month follow-up. The efficacy and cost-effectiveness of the two interventions will be assessed. Treatment outcomes will comprise cancer-specific quality of life (primary outcome), anxiety and depressive symptoms, psychological flexibility, self-compassion, health-related quality of life, resource use, and intervention’s acceptability and feasibility. Study 1 will also include immunological and epigenetic markers associated with breast cancer prognosis and mental health. Outcome assessors will be blind to group allocation. Statistical analyses will be conducted using an intention-to-treat approach. Analyses of moderators and mediators of change will also be performed.

**Discussion:**

These trials examine the efficacy and cost-effectiveness of an integrative ACT and compassion-based intervention tailored for women with breast cancer. Greater improvements in psychosocial, biological and resource use are expected in the Mind group, when compared to the control group(s). Results will likely support the potential benefits of the Mind programme for breast cancer patients and highlight the clinical relevance of integrative and holistic interventions in oncology.

**Trials registration {2a, 2b}:**

ClinicalTrials.gov NCT05642897 and NCT06212414. Registered on December 8, 2022, and January 18, 2024.

**Supplementary Information:**

The online version contains supplementary material available at 10.1186/s13063-024-08626-4.

## Introduction

### Background and rationale {6a}

Breast cancer (BC) is the most diagnosed cancer in women, with millions of new cases each year worldwide [1]. The disease and respective treatments lead to physical and psychological impairments, which may contribute to depression, anxiety, fatigue, sleep disturbances, and altered body image and quality of life [2, 3]. Due to improved medical treatment, cancer patients’ survival rates and longevity are increasing and, therefore, the long-term psychological and physical outcomes, and its treatments, are more pervasive [4]. After treatment, cancer patients usually experience a lack of continuity, guidance and resources [5] that impair the transition to survivorship [6].

Psychological distress in BC are associated with increased functional impairment, poor treatment adherence [7] and decreased survival [8]. Depression, for instance, may accelerate cancer progression through endocrine and immunological changes [9], particularly chronic inflammation, with increased production of interleukin-6 (IL-6), C-reactive protein and TNFalpha [9]. Cancer progression in depressed patients may be due to reduced cellular immunity, which is responsible for eliminating cancer cells [10]. MicroRNAs (miRs), an emerging tool in cancer epigenetics, have been shown to influence mental health and BC [11]. In both contexts, some miRs seem to constitute good predictors of psychological and physical changes. Interestingly, many of these miRs, namely miR-21, miR-146a, miR-155 and miR-Let-7, also regulate inflammation, a process with a significant impact on BC progression and recurrence, as well as psychopathology [11, 12].

The variables that explain the development and maintenance of psychopathology in BC appear to be psychological rather than cancer-related [13], which is consistent with the idea that one’s relationship with adverse experiences is an important determining factor of their impact on well-being [14]. These findings highlight the need for developing and testing the efficacy of psychological interventions for women with BC to foster adaptive emotion regulation strategies that promote women’s mental health, and that empowers and prepares them for survivorship. These interventions may be particularly relevant during radiotherapy treatment, when BC patients remain under treatment while preparing to return to work and their usual daily routines [15].

Interventions that use mindfulness have been designed to improve BC patients’ ability to cope with their disease. Several meta-analyses have consistently found effects in decreasing stress, depression and anxiety, yet indicating that further research is needed to understand their clinical significance [16, 17]. Mindfulness appears to lower inflammation markers (such as TNF and IL-6) [18] and restore immune function, and seems to result in epigenetic benefits in women with BC [19], although no study has yet explored the impact of such interventions on miRs in this population.

Another promising intervention approach that incorporates mindfulness is Acceptance and Commitment Therapy (ACT) [14]. ACT seems to be particularly pertinent for the cancer context [20], especially given that BC patients may present relevant levels of experiential avoidance (e.g. denial, cognitive distraction) and lack of committed action (e.g. lack of adherence to treatment plans, not engaging in self-care) [21], two key processes that ACT aims to target. Nevertheless, ACT trials on BC are limited and more research is needed [22].

Concomitantly, a growing body of evidence demonstrates the importance of developing a self-compassionate attitude after a cancer diagnosis. The application of Compassion-Focused Therapy (CFT) [23] has received increasing attention and has presented positive effects on mental health outcomes (see [24] for a review). Self-compassion is associated with decreased body dissatisfaction and psychological distress in BC [25]. Furthermore, self-compassion is suggested to be protective against stress-induced inflammation and inflammation-related diseases [26].

Having several common features, ACT and compassion-based approaches are considered to be complementary and compatible contextual behavioural interventions. Their integration has been the focus of international attention, particularly by the current research team, which has disseminated promising findings on this integration in completed [27, 28] and ongoing (LIFEwithIBD; iACTwithPain; eBefree) research projects. Given the potential relevance of these approaches in improving well-being in cancer populations, and the need to develop and test the efficacy of psychological interventions for BC patients, the Mind programme for cancer patients was previously developed [29]. This manualised group intervention was developed by integrating ACT and compassion components specifically adapted to a cancer population. A recent pilot study presented preliminary findings on this intervention [29], suggesting high acceptability and efficacy in improving self-reported psychological health. Nevertheless, the small sample size (*N* = 32), methodology (inactive control group) and exclusive reliance on self-reported data limit the interpretation and generalisation of results, creating an avenue for the optimisation and further testing of the programme through more robust methods.

### Objectives {7}

Both randomised controlled trials (RCT) presented in this manuscript aim to determine the efficacy (aim 1) and cost-effectiveness (aim 2) of an optimised version of the Mind programme, an integrative ACT and compassion-based intervention tailored for women with BC, compared to an active (i.e. support group) and/or inactive (i.e. treatment as usual group) control condition. These trials also aim to determine the contribution of mediators (e.g. psychological flexibility) and moderators (e.g. age) factors in treatment effect (aim 3). Efficacy indicators comprise cancer-related quality of life (primary outcome), anxiety and depressive symptoms, psychological flexibility, self-compassion, health-related quality of life, and intervention’s acceptability and feasibility (secondary outcomes). Cost-effectiveness indicators cover participants’ costs and resource utilisation outside the hospital setting (secondary outcome). Immunological and epigenetic markers (secondary outcomes) will also be considered (study 1).

The following hypotheses will be tested: (1) women with BC receiving the Mind programme will show increased levels of psychosocial and biological outcomes than those in the control group(s); (2) the Mind group will demonstrate superiority in a cost-effectiveness analysis compared to the control group(s); (3) changes in specific secondary outcomes (e.g. psychological flexibility) will mediate treatment effects on women cancer-related QoL with some characteristics (e.g. older, educated).

### Trial design {8}

**Fig. 1:**
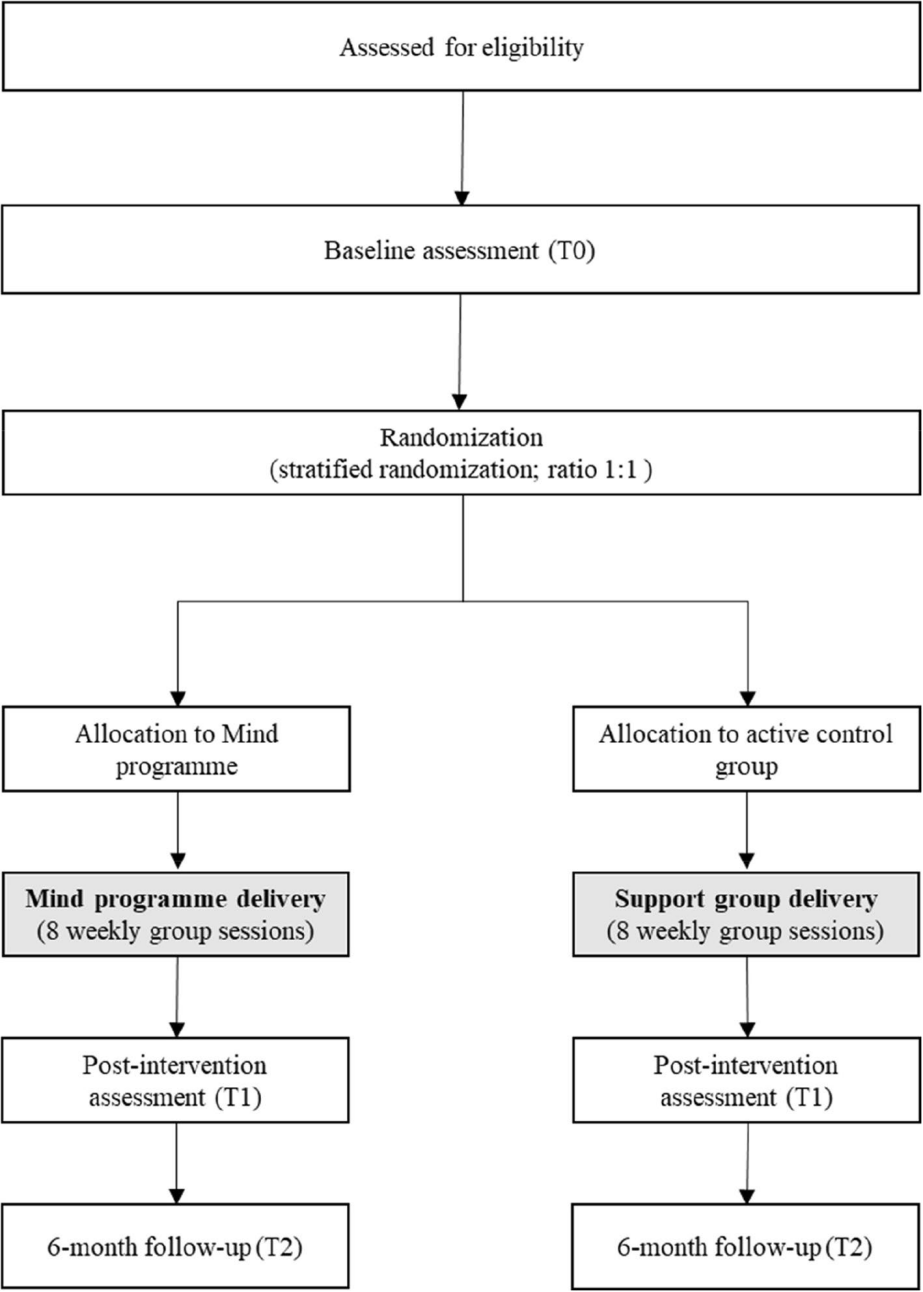
Flow chart for study 1

**Fig. 2:**
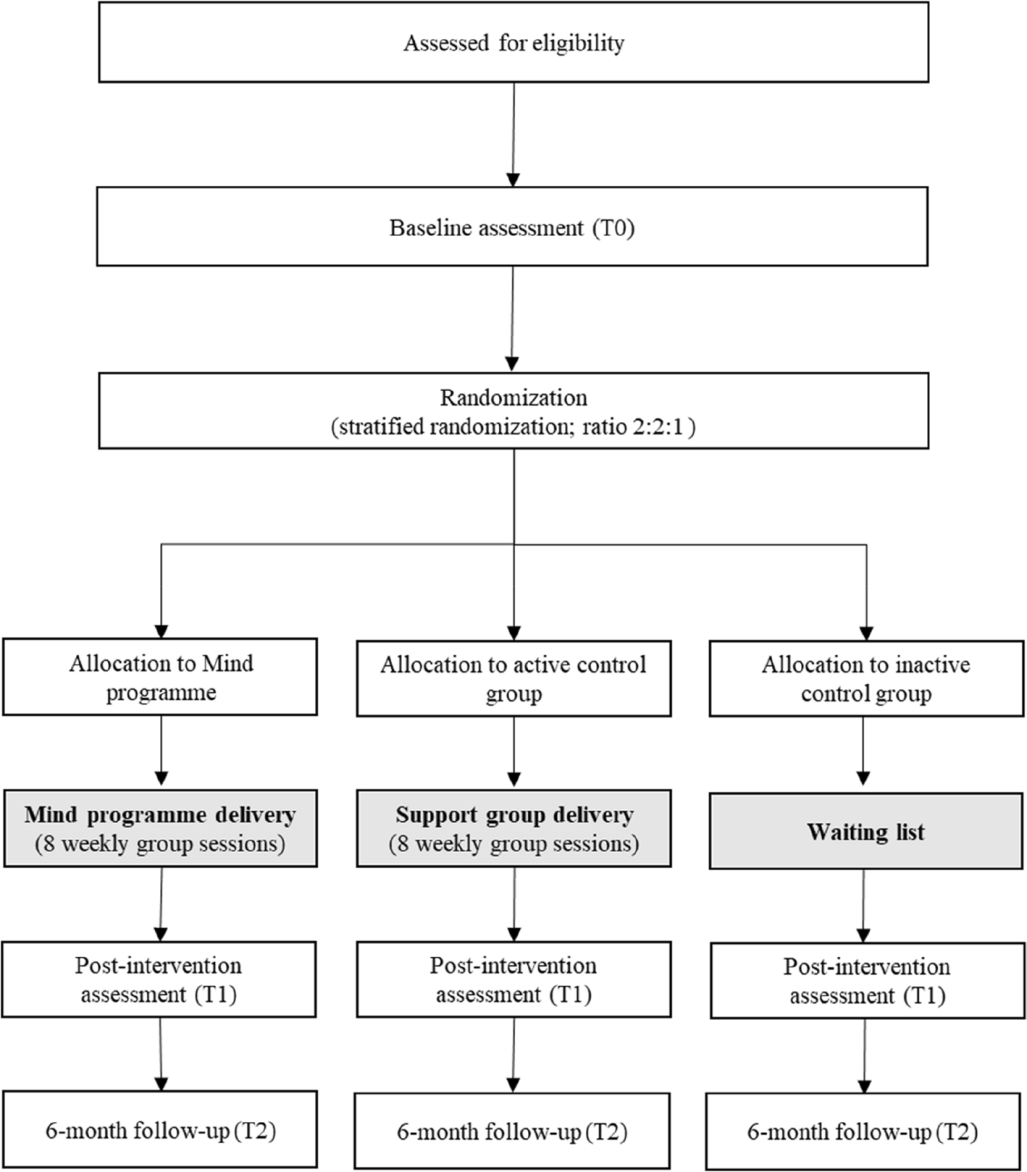
Flow chart for study 2

This research uses a RCT (study 1) with parallel 1:1 assignment to two study arms (Mind programme vs. active control) and a RCT (study 2) with parallel 2:2:1 assignment to three study arms (Mind programme vs. active control vs. inactive control). A 2:2:1 ratio was chosen for study 2 for ethical considerations—to reduce the number of participants who delay receiving potentially beneficial treatments, while still allowing a comparison group to measure intervention effects. The decision for conducting two different trials was taken several months after the first trial’s start and the understanding that the recruitment rate was lower than needed to conduct intervention groups at a regular pace, for reasons beyond the team’s control. The adoption of a different design (3-arm instead of 2-arm) was related to the opportunity of achieving a more robust design with the second RCT (study 2). Figures 1 and 2 provide the recruitment flow and condition allocation for each study.

## Methods: participants, interventions and outcomes

### Study setting {9}

Both trials are being conducted in Portugal. The research team is based at Coimbra Hospital and University Centre, where participants are being recruited (study 1). For study 2, participants are being recruited nationally and online, through social media outlets, newsletters and relevant community groups (as described in Sect. 15). Participant data will be collected in person (study 1) or electronically through secure online survey tools (e.g. LimeSurvey) (study 1 and study 2).

### Eligibility criteria {10}

Inclusion criteria for both studies (except when stated otherwise): (a) age between 18 and 70; (b) primary diagnosis of BC (stages between I and III); (c) undergoing radiotherapy treatment at CHUC (study 1); or having a scheduled radiotherapy treatment starting within 2 months, currently undergoing radiotherapy treatment or having finished radiotherapy treatment not more than 6 months prior, at any hospital in Portugal (study 2); (d) able to understand and answer to self-report questionnaires in Portuguese; (e) having access to a computer, tablet or smartphone with internet (study 1 and study 2).

Exclusion criteria for both studies: (a) currently undergoing any form of psychological intervention; (b) current diagnosis of severe psychiatric illness (psychotic disorder, bipolar disorder, substance abuse and personality disorder) or suicidal ideation; (c) diagnosis of neurological disease. Women who present suicidal ideation will be referred to psychological and/or psychiatric services for further support.

### Who will take informed consent? {26a}

If women are interested and eligible to participate (assessed through a screening interview in person, in study 1, or conducted via phone call, in study 2), a member of the team will obtain their written (study 1) or electronic (study 2) informed consent.

### Additional consent provisions for collection and use of participant data and biological specimens {26b}

In study 1, blood samples will be collected at the Radiotherapy Service of CHUC, with the collaboration of the Service staff and only after obtaining patients’ written informed consent for collection and analysis of their biological specimens and data. Study 2 does not involve the collection of biological specimens.

## Interventions

### Explanation for the choice of comparators {6b}

Both studies include two intervention groups (see Sect. 11a, below): the Mind programme group and the support group. The Mind programme was previously developed and tested and presented good preliminary findings [29]; the comparator is a support group intervention similar to those implemented in prior RCTs conducted in BC patients [30]. The purpose of this comparator group is to control for effects related to group belonging, personal sharing of experiences, and contact with therapists and fellow participants.

A second comparator (inactive control group) is also included in study 2. Both comparators in this study allow time effect control and treatment allocation blindness.

Regardless of the intervention assigned to each participant, all patients will continue receiving the recommended medical treatment for their clinical diagnosis.

### Intervention description {11a}

#### Mind programme for women with BC (optimised version)

The Mind programme is a manualised psychological intervention tailored to women with BC and intended to be delivered by two licenced psychologists, in a face-to-face or online group format. This intervention comprises 8 weekly group sessions, lasting 90–120 min each. Its content was optimised by the research team based on (a) the intervention’s pilot study results [30]; (b) the research team’s experience in psycho-oncology and/or in delivering similar interventions to patients with chronic medical conditions; (c) previous clinical experience in delivering the Mind programme in its pilot study; and (d) previous clinical experience in delivering the LIFEwithIBD programme, an adaptation of the Mind programme for people with inflammatory bowel disease [31, 32] As part of optimising the intervention (Table 1), modules on shame and self-criticism were improved, and the forgiveness module of the original Mind programme was replaced with a module on gratitude. Some compassion strategies (e.g. Compassionate Image exercise) were replaced by Mindful Self-Compassion practices, and the module on the observing self, as conceptualised by ACT, was removed [33]. Therefore, the optimised Mind programme (Table 1) comprises the following core themes: (a) acceptance of internal experiences, willingness, values and committed action [34]; (b) mindfulness practice [35]; (c) compassion-based practices [e.g. Mindful Self-Compassion [33], Compassion-Focused Therapy [23] and Loving Kindness meditation [36]; and (d) gratitude (towards one’s body) [36]. Educational aspects of physical and mental fatigue were also included. Each session will follow a similar format: (1) meditation practice; (2) discussion about between-session assignments (homework); (3) presentation of the session’s topics and in-session experiential exercises and practices; and (4) mindfulness or compassion meditation practices. Participants will have access to a participants’ manual (including handouts and audio files), which will guide them over the sessions and exercises. The between-session activities (e.g. mindfulness exercises and compassion practices, available through audio files made available through the sessions) are in line with the topics covered in each session and are prescribed to participants for daily practice until the next session. Participants with two missed sessions will be considered intervention non-completers, but they will be allowed to maintain their participation (and included in intention-to-treat analyses). To ensure treatment integrity, the following will be assured: (a) the Mind programme will be delivered in a co-therapy system, by two therapists with (at least) a Portuguese psychologist licence, a MSc degree in Clinical Psychology, and experience and training in ACT, mindfulness and compassion-based interventions; (b) all therapists will follow the intervention manual; (c) by the end of each session, the co-therapist will complete a checklist of the contents discussed in each session to safeguard treatment fidelity.

**Table 1:** Original Mind programme vs. optimised Mind programme

Session	Mind programme (original version from pilot study; Trindade et al., 2020)		Optimised Mind programme	
	Core themes	Components	Core themes	Components
1	Introduction to the programme	- Introduction and orientation to the programme - Participants’ self-presentation - Identification of areas of suffering and promotion of creative hopelessness - Introduction to mindfulness - A first taste of mindfulness: eating a raisin - The importance of practising informal mindfulness daily	Welcoming and creative hopelessness	- Introduction and orientation to the programme - Participants’ self-presentation - Identification of areas of suffering and promotion of creative hopelessness - Introduction to mindfulness - A first taste of mindfulness: eating a raisin - A first mindfulness formal practice: 3-min breathing space - The importance of practising informal mindfulness daily
2	Body awareness	- Psychoeducation about emotion regulation (evolutionary basis of emotions) - Becoming aware of our bodily states (mindfulness of breathing and body scan)	Body	- “Notice 5 things” exercise - Evolutionary perspective of the functioning of the mind (“Yellow jeep” exercise) - Mind–body interactions - Adaptive functions of internal experiences and their physiological consequences - Promoting awareness of physical states and promote emotion regulation through the body (body scan)
3	Cognitive defusion and values clarification	- Promoting cognitive defusion - Mindfulness of sounds and thoughts - Life values identification - Committed action (passengers on a bus metaphor; surprise birthday party exercise; bull’s eye exercise)	Clarification of values	- Clarification and definition of life values (passengers on the bus metaphor; surprise birthday party) - Individual assessment of the consistency between one’s values and current behaviour
4	Compassion I	- The importance of practising informal mindfulness (3-min breathing space) - We all need compassion - Cultivating compassion—short Loving Kindness meditation	Values/committed action and self-care	- Identification of objectives and obstacles to committed action (bull’s eye exercise) - Education on fatigue: strategies to cope with different types of fatigue (physical and mental fatigue; self-care)
5	Acceptance	- Understanding acceptance and its determinant role to well-being - Controlling is the problem - Discussion about the unfair social pressure cancer patients usually feel to be hopeful and positive - Promoting acceptance and willingness (taking your mind for a walk exercise; physicalising exercise; inviting a difficulty exercise)	Compassion	- Introduction to the concepts of suffering, shame and body image - Introduction to compassion as a strategy to manage difficult feelings and general suffering; tacking self-criticism with self-kindness - Self-compassion and relaxation training (soothing rhythm breathing and Loving Kindness)
6	Self-care and self as context	- Promoting self-care: how to deal with fatigue (differences in physical fatigue and mental fatigue) - Self as context exercises (“How old is this problem exercise”; observing self exercise)	Acceptance/cognitive defusion	- The power of thinking: understanding the functioning of the mind and cognitive defusion promotion (“Thinking of an apple” exercise; “I am having the thought that I am…” exercise) - Promotion of acceptance and willingness through cognitive defusion (inviting a difficulty exercise)
7	Compassion and forgiveness	- Strategies to cultivate a compassionate mind (complete Loving Kindness meditation; Compassionate Image exercise) - The importance of forgiveness: forgiveness mediation (asking for forgiveness, forgiving ourselves and forgiving others)	Compassion and gratitude towards the body	- Continuation of compassionate training: developing compassion for oneself and for others (Loving Kindness) - Gratitude meditation - Promotion of compassion and gratitude towards our body (reassuring touch exercise)
8	Committed action	- Further development of engaged living (gardening metaphor) - Identification of obstacles to committed action and strategies to overcome them - End of programme discussion	Committed action/programme summary	- Engaging in committed action: strategies to overcome expected obstacles (the gardening metaphor; identification of probable future obstacles and how to overcome them) - Summary of the intervention: how the promoted psychological processes are connected interact with each other

Note: All sessions, from session 2, start with a mindfulness/self-compassion exercise

#### Support group intervention

This intervention (comparator) comprises 8 weekly 90 to 120-min sessions intended to be delivered by two licenced psychologists, in a face-to-face or online group format. Table 2 presents the BC-related content of the intervention, which will be discussed among participants in each session. The facilitators of this intervention will engage in a purely moderating role (e.g. facilitators should guarantee that all the participants have the chance to participate, and no one will use that time extensively; facilitators should also intervene if a participant states inaccurate information). As it is the case with the Mind programme group, participants with two missed sessions will be considered intervention non-completers, but they will be allowed to maintain their participation (and included in intention-to-treat analyses).

**Table 2:** Session content for the support group intervention

Session 1	Participant self-introduction, group rules and discussion of expectations related to the intervention
Session 2	Emotional impact of BC diagnosis
Session 3	BC treatment side effects
Session 4	BC impact on family and caregivers
Session 5	Work-related and physical impact of BC (e.g. fatigue)
Session 6	Body image and sexuality after BC
Session 7	Self-care strategies
Session 8	Conclusion and final remarks

#### Treatment as usual (waiting list)

The inactive control group (study 2) will receive the usual treatment for cancer patients in Portugal. In this country, the public healthcare system does not offer psychological counselling or psychotherapy as part of the regular care of women with BC. To gain access to this service, a referral from a physician is needed and not always granted due to lack of resources. At the end of this project, the intervention that proves to be most efficacious will be offered to the participants allocated to this condition.

### Criteria for discontinuing or modifying allocated interventions {11b}

The discontinuation or modification of the interventions is not expected before the trials’ closure due to unlikely adverse effects. Nevertheless, if they occur a standardised procedure will follow (see Sect. 22). Participants may opt out of the intervention or the studies at any time.

### Strategies to improve adherence to interventions {11c}

Adherence during the treatment phase will be monitored as follows: (a) Participants will receive a phone call from a team member providing all the important information (e.g. sessions schedule and calendar, local/link where the intervention takes place, facilitators’ names and contacts), followed by an email with the same details. (b) They will also receive a reminder of the upcoming session by email a few hours before the meeting and by phone call/text message just before the session. (c) Materials for between-session assignments (homework) will be sent by email immediately after each Mind programme session and a reminder reinforcing at-home practice will follow through the same channel 2–3 days after.

### Relevant concomitant care permitted or prohibited during the trial {11d}

Women receiving any form of psychotherapy at screening are not eligible. However, participants are not prohibited from seeking other interventions or treatment during their participation in the trials. Engagement in any other intervention or treatment must be reported to the research team during their participation.

### Provisions for post-trial care {30}

Adverse events are not expected (see Sect. 22). Therefore, there are no provisions for any additional post-trial care or to provide compensation to those who suffer harm from trial participation.

### Outcomes {12}

The primary outcome in both studies is cancer-specific quality of life [2]. Likewise, secondary outcomes in both studies are anxiety and depressive symptoms, psychological flexibility, self-compassion, health-related QoL, resources consumption, and intervention’s acceptability and feasibility. Study 1 also includes as secondary outcomes immunology [inflammatory biomarkers formerly associated with depression and anxiety (e.g. CRP, IL-6 and TNFalpha); biomarkers of the development of effective immune surveillance (e.g. IFNgamma, IL-12/18, GM-CSF); suppressive cytokines that may block the development of effective anti-tumour immune responses (e.g. IL-10, IL-4/13)] and epigenetics markers [expression of miRs associated with stress response, inflammation or BC prognosis (miR-21, miR-146a, miR-155 and miR-Let7)]. Additional variables (e.g. sociodemographic and clinical variables, major life events, psychological processes awareness, at-home practice frequency, efficacy expectancy) will be used as covariates or moderating variables if significantly correlated with primary and secondary outcomes. Most outcomes will be assessed at baseline, lead statisticians, and at 6-month follow-up period. See Sect. 18a and Table 3 for further details.

**Table 3:**
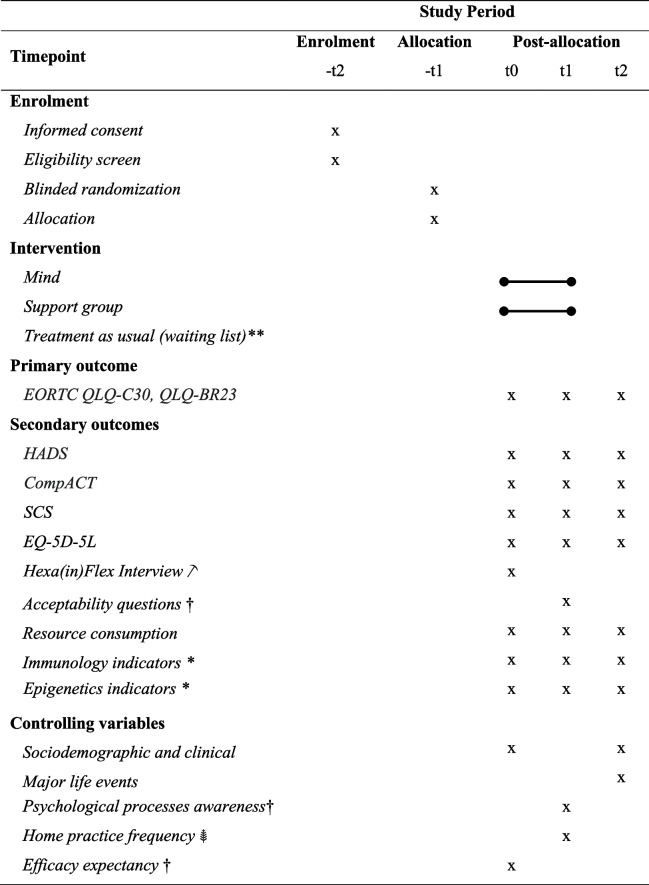
Schedule of enrolment, intervention and assessments for study 1 and study 2

Note. EORTC QLQ-C30, QLQ-BR23, The European Organization for Research and Treatment of Cancer Quality of Life Questionnaire and its Supplementary Questionnaire Breast Cancer Module, HADS, The Hospital Anxiety and Depression Scale, CompACT, Comprehensive Assessment of Acceptance and Commitment Therapy processes, SCS, Self-Compassion Scale, EQ-5D-5L, health-related quality of life

**only for study 2 participants

*only for study 1 participants

only for a subsample of study 1 participants

†only for intervention groups

only for Mind participants

### Participant timeline {13}

See Table 3.

### Sample size {14}

Regarding study 1, power was considered according to a sensitivity analysis of the likely attainable sample size and compare with the effect sizes estimated by previous meta-analyses and studies suggesting improvements in mood and QoL between SMD = 0.2 to 0.6, favouring psychotherapies over controls [15, 16]. It is anticipated that up to *N* = 150 (*n* = 75 per group) participants will be recruited, and attrition rates of between 10 and 30% can be expected. The planned analysis specifies a repeated-measures linear mixed models analysis, a group allocation of 1:1, alpha of 0.05, a minimum of 80% power, three assessment points and a compound symmetry correlation matrix (rho = 0.5; Lu et al., 2008). Under these parameters, the study is powered to reliably detect a between-group difference on cancer-specific QoL at follow-up of SMD = 0.51 (WebPower; [37]).

For study 2, the GLIMMPSE software was used to calculate the minimum sample size required for a three-armed linear mixed model with three measurements. Assuming a comparable effect size to that estimated in study 1 (SMD = 0.5) favouring the Mind intervention group over waiting list at 6 months for QoL, an unstructured correlation matrix, a group allocation of 2:2:1, alpha of 0.05 and a minimum of 80% power, the minimum sample size required is *N* = 117. To account for up to 30% attrition, this sets the minimum total sample size at *N* = 153 [38].

### Recruitment {15}

In study 1, women being treated for BC in the Radiotherapy Service of CHUC will be invited to participate. The point of recruitment will be at the beginning of radiotherapy treatment. The trial will be introduced to potential participants by their radiotherapy physician. A screening interview will be conducted with interested patients to assess eligibility and obtain written informed consent.

In study 2, the trial will be disseminated among the Portuguese population via press releases, social media platforms (e.g. postings with relevant groups and communities via Facebook and Instagram) and BC patients’ associations electronic distribution lists. Women with BC will register their interest by completing a form hosted on a secure online platform, which can be easily found in the project’s website. Alternatively, interested persons can contact research staff via email provided in the study advertisements. A screening interview, conducted by phone call, is later conducted to assess eligibility and obtain electronic informed consent.

## Assignment of interventions: allocation

### Sequence generation {16a}

Computer-based randomisation will be used (www.random.org/lists/), via a numbered sequence, in both studies, by blinded researchers. Allocation of women who underwent chemotherapy and those who did not will be proportionate among the groups (stratified randomisation).

### Implementation {16c} and concealment mechanism {16b}

After sample selection and filling of the baseline measures, participants will be randomly allocated to one of two (study 1) or three (study 2) conditions: (a) experimental group (Mind programme), (b) active control group (support group) and (c) inactive control group (treatment as usual; study 2 only). Allocation and assignment of participants will be conducted by members of the research team who do not have access to participants’ information other than study identification code and chemotherapy status.

## Assignment of interventions: blinding

### Who will be blinded {17a}

Participants, outcome assessors and data analysts will be blind to treatment assignment. Blinding will be made possible through: (a) providing participants essential information regarding the treatment, without compromising its content concealment, and using the same format to deliver different treatments (see Sect. 11a); (b) assessing outcomes via data collection platform (see Sect. 18a); and (c) restricting access to participants’ treatment assignments in the study database only to staff whose role is to contact participants before and throughout the intervention phase. It is not impossible for participants to deduce their treatment group based on the content they receive.

### Procedure for unblinding if needed {17b}

Staff members whose role is to contact participants before and throughout the intervention phase will not be blinded to the treatment assignment. In the unlikely event that an adverse event should occur, unblinding of other members and of participants can take place.

## Data collection and management

### Plans for assessment and collection of outcomes {18a}

Outcome self-report measures will be completed independently by participants, reducing risk of bias. These measures will be hosted on LimeSurvey, a secure web-based data collection platform. A description of all research measures follows (see Table 3 for assessment timing).

### Primary outcome

#### Cancer-specific QoL

The European Organization for Research and Treatment of Cancer Quality of Life Questionnaire (EORTC QLQ-C30; [39]; Portuguese version: [40]) is a 30-item questionnaire that reflects the multidimensionality of the QoL construct, comprising five functional subscales (physical, role, cognitive, emotional and social); a global health/QoL subscale; three symptom subscales (fatigue, pain and nausea/vomiting); single items for the assessment of additional symptoms commonly reported by cancer patients (dyspnoea, appetite loss, sleep disturbance, constipation and diarrhoea); and one more item related to the perceived financial impact of cancer and cancer treatment. All the items are scored on a 4-point Likert type scales ranging from 1 (not at all) to 4 (very much), except the two items of the global health/QoL subscale, which use a modified 7-point linear analogue scale. The 23-item Supplementary Questionnaire Breast Cancer Module (QLQ-BR23) additionally assesses body image, sexual function, sexual enjoyment and future perspective. In the Portuguese validation study, global QoL presented a Cronbach’s alpha of 0.88 and the different subscales presented Cronbach’s alpha ranging between 0.57 and 0.88 [41].

### Secondary outcomes

#### Anxiety and depressive symptoms

The Hospital Anxiety and Depression Scale (HADS; [42]; Portuguese version: [43]) is a 14-item self-report questionnaire that includes two subscales, one measuring anxiety and one measuring depressive symptoms, which are scored separately. Each item is rated on a 4-point scale from 0 (no impairment) to 3 (severe impairment). In the Portuguese validation study, the anxiety and depression subscales had Cronbach’s alpha values of 0.76 and 0.81, respectively.

#### Psychological flexibility

The Portuguese Comprehensive Assessment of Acceptance and Commitment Therapy processes (CompACT; [44]; Portuguese version: [45, 46]) is a measure of psychological flexibility, as conceptualised by ACT. Items are rated on a 7-point Likert scale from 0 (never true) to 6 (always true). The Portuguese validation of the CompACT in illness resulted in an 8-item version.

#### Self-compassion

The Self-Compassion Scale (SCS; [47]; Portuguese version: [48]) is a 26-item instrument with six subscales to assess self-compassion: self-judgement, self-kindness, overidentification, mindfulness, isolation and common humanity. Items are rated on a 5-point Likert scale from 1 (almost never) to 5 (almost always). The Portuguese study of the SCS presented a Cronbach’s alpha of 0.94 for the total scale, and alphas between 0.70 and 0.88 for the subscales.

#### Health-related QoL

The EQ-5D-5L ([49]; Portuguese version: [50]) is a generic instrument for assessing health-related QoL in economic evaluation. It is a self-report questionnaire with five items/dimensions (mobility, self-care, usual activities, pain/discomfort and anxiety/depression) and one visual analogue scale to assess self-rated health status. For each dimension, participants select one of five levels: 1 (no problems), 2 (slight problems), 3 (moderate problems), 4 (severe problems) and 5 (extreme problems). Scores will be converted into utility weights using the value set estimated for Portugal to obtain a single summary index ranging from 0 (death) to 1 (perfect health). In addition, the visual analogue scale records the patient’s self-rated health state, between 0 (the worst health you can imagine) and 100 (the best health you can imagine). In the Portuguese validation study, the EQ-5D showed a Cronbach’s alpha of 0.72.

#### Resource use

Resource use related to BC diagnosis and treatment will be collected via an adapted version of the UK Cancer Costs Questionnaire (UKCC) Version 2.0 [51]. The UKCC collects data on the number of contacts with health professionals outside the hospital setting (general practitioner, nurse, as well as other medical and non-medical specialities), the number of emergency room visits and the medication taken. Other non-medical resources incurred by the family, such as the use of personal social services to help in daily activities, travel and transportation costs, as well as other costs related to a diverse array of goods and services the patient has used to deal with BC, are also collected by the questionnaire. In addition, the questionnaire has a section devoted to collecting data about the time taken off work due to BC, which will be used to estimate productivity losses.

*Immunology and epigenetics markers* (study 1 only). Blood samples will be collected, stored and analysed as stated in Sect. 33.

*ACT processes* (subsample of study 1 only). Hexa(in)Flex Interview is a semi-structured interview that aims to assess qualitatively the subjective experience of the six core processes of the Psychological (In)Flexibility Model underlying ACT, in women with BC. This interview was developed within the context of this project. It comprises two parts: (a) an introduction to the aims of the interview, as well as introductory questions regarding diagnostic information (e.g. duration of diagnosis, treatment phase, support network) and general coping and adaptation to the cancer diagnosis; (b) five sections of open questions aiming to assess experiential avoidance versus acceptance, cognitive fusion versus defusion, conceptual versus contextual self, past and future conceptualised (auto-pilot) versus contact with present moment, and lack of values clarity and action versus commitment to valued action. Each section has instructions on how the interviewer should conduct the questioning, as well as additional tips and caveats that should be considered.

#### Acceptability of the intervention

At the end of the intervention, participants will complete a final evaluation form of the programme assessing several aspects, including the match between their expectations and the actual intervention, their overall level of satisfaction with the programme, the perceived usefulness of the intervention to help them cope with their problems and solve their difficulties, and their intention to recommend or use the programme in the future if necessary, among other aspects.

#### Feasibility of the intervention

It will be measured through participants’ adherence, including the number of sessions attended, the proportion of treatment completers (defined as those who participated in at least 75% of the sessions) and the number of homework practices completed between each session. Dropout rates (i.e. the proportion of participants that dropped out from the intervention before completing it) will also be considered as an indicator of feasibility.

### Additional variables

#### Sociodemographic and clinical variables

Variables such as age, education level, marital status, residency area, household composition and income, BC diagnosis, BC stage, TNM classification, time since BC diagnosis, immunohistochemical data, values of CA15.3, haemoglobin, leukocytes, and platelets, fertility preservation, previous chemotherapy treatment (and number of cycles), use of psychopharmacological medication, and relevant clinical history will be collected.

#### Major life events

The Major Life Events Questionnaire (MLEQ; [52]) assesses the occurrence of major life events in the previous 12 months. The MLEQ was based on the Psychological Stress Index [53] and comprises 22 items that represent possible major life events (e.g. marriage, divorce, pregnancy, interpersonal conflict, death of a significant other). For each item, participants report the occurrence or non-occurrence of a specific event during the previous 12 months.

#### Psychological processes awareness

The Awareness of Contextual Therapies Processes Scale was developed by the authors of these studies to assess the extent to which participants increase their awareness of the psychological processes and mechanisms involved in the intervention.

#### Home practice frequency

At each session of the Mind programme intervention, participants will be asked about whether they had completed the at-home practice that was assigned in the previous session.

#### Efficacy expectancy

Participants will answer the following yes–no single question about the efficacy expectancy at baseline: “Do you believe that the intervention will bring benefits to your quality of life and mental health?”.

## Plans to promote participant retention and complete follow-up {18b}

Outcome self-report measures will be completed online so that participants may access them at convenient times and places. Participants will receive automated assessment survey invitations and reminders. Dedicated staff members (see Sect. 19) will review participants’ responses and send reminders by email or text if assessment surveys are not complete, until the completion of the assessment period. A database checklist will be available to ensure that all measures are being collected according to the schedule. Participants will also receive a 25€-worth gift voucher for their full participation, at the 6-month follow-up.

Irrespective of intervention discontinuation or deviation from protocol, outcomes data collection will continue for all participants. A variable representing the extent of treatment completion (number of sessions attended) will be added, and sensitivity analyses to evaluate its impact on intervention outcomes will be performed.

### Data management {19}

Data management will be performed in accordance with the European regulations for data processing [54] and the University of Coimbra’s guidelines [55]. There are designated team members to enter, code, protect and store data. During the course of the project, these researchers will enter and code information, collected throughout recruitment, intervention and assessment phases, in a statistical analysis software file using unique participant IDs to identify participants. These researchers will have access to participants’ identifiable data and safeguard it in a secure password-protected local drive. They will also be responsible for preparing database checklists and alerts, sending reminders, monitoring data irregularities (e.g. skip patterns, out of range data, completion times), sharing de-identified data with lead statisticians and granting access to authorised entities conducting trial-related monitoring, audits and inspections. Measures scoring will be done via syntax to minimise errors. After project termination, data, stored in a secure password-protected local drive, will be kept for 5 years, before disposal.

### Confidentiality {27}

Personal information about potential and enrolled participants will be collected during the screening interview (face to face or via phone call) and stored in a secure password-protected local drive (see Sect. 19). The database containing research records will use unique participant ID to promote data sharing. The primary source of data will come from self-reported measures hosted on LimeSurvey, a secure web-based data collection platform. Only designated team members will have access to participants’ identifiable data. De-identified data will be shared on need and solely among the research team.

### Plans for collection, laboratory evaluation and storage of biological specimens for genetic or molecular analysis in this trial/future use {33}

Blood samples will be collected in vials with no anti-coagulation factors, at the Radiotherapy Service of CHUC, with the collaboration of the Service. All patients will be asked for the presence of infection symptomatology (e.g. flu, cold) at the blood collection time and on the previous 15 days. Samples will be maintained at 4 °C until and during transport to the Laboratory of Molecular Pathology (at Portuguese Institute for Oncology at Coimbra Francisco Gentil) where they will be processed. Sample processing involves serum collection through centrifugation, which is performed within 1 h (maximum 2 h, but these situations are documented) after blood collection. Serum samples will be stored at − 80 °C until analysis. Cytokines will be quantified by flow cytometry at the Molecular Pathology Laboratory and epigenetic studies will be performed at Institute of Molecular Pathology and Immunology of the University of Porto, through quantification of microRNAs. After project termination, samples and data will be safely stored for 5 years and then disposed of. Their future use in ancillary studies is not yet planned.

## Statistical methods

### Statistical methods for primary and secondary outcomes {20a}

#### General analytic procedures

The superiority of the Mind programme over the control group(s) will be tested through the analysis of changes in the primary (cancer-specific QoL) and secondary outcomes (anxiety and depressive symptoms, psychological flexibility, self-compassion, health-related QoL, resources consumption), controlling for sociodemographic and clinical variables and potential moderation effects of variables such as the awareness of psychological processes, the frequency of at-home practice, the number of major life events occurring during the previous year and efficacy expectancy. The Mind programme will be compared against the support group in study 1 and against both the support and treatment as usual (waiting list) groups in study 2. Study 1 also includes immunological and epigenetic markers as secondary outcomes. The analytical approaches for both studies are otherwise comparable.

#### Analyses by aims

Aim 1: Determine the efficacy of the Mind programme compared to an active (i.e. support group) and/or inactive (i.e. treatment as usual group) control condition at post-intervention and at 6-month follow-up period.

The planned analysis for each outcome is a repeated-measures linear mixed model, including polynomial simple slopes to identify non-linear change over time. Within-group and contemporaneous between-group post hoc comparisons, using independent- or paired-samples *t*-tests (as appropriate), adjusted for multiple comparisons using the modified Bonferroni adjustment, will be performed. The efficacy of the intervention can be inferred through the evaluation of the simple slopes for each treatment condition, whereby the experimental condition demonstrates a significant improvement in QoL scores over time, and this improvement exceeds that of the control condition. The clinical significance of changes presented by each participant will be determined by computing the reliable change index (RCI) and considering mixed models of repeated measures (i.e. growth modelling analysis). In the former, we will calculate primary or secondary outcome RCI for each participant between pre- and post-intervention, as well as pre-intervention and 6 months. An RCI of ± 1.96 is considered statistically significant at *p* = 0.05, and the proportions of participants within each treatment condition who demonstrate significant improvement, decline and no change will be recorded. Fisher’s exact test will be used to examine for significant differences in the proportion of change between treatment allocations. Finally, we will perform correlation analysis between the raw RCI values and demographic variables separately for each treatment condition. This final analysis is used to examine potential predictors of individual change and the nature of that change.

Aim 2: Determine the cost-effectiveness of the Mind programme compared to an active (i.e. support group) and/or inactive (i.e. treatment as usual group) control condition at post-intervention and at 6-month follow-up period.

The within-trial economic analyses will be carried out in line with standard health economic methods, following the Consolidated Health Economic Evaluation Reporting Standards (CHEERS) checklist [56]. An economic evaluation of each trial will be conducted. Each economic evaluation will include a cost-utility analysis using the outcome quality adjusted life years (QALYs). EQ5D-5L utility scores will be used to estimate QALYs over each trial period using the area under the curve method [57]. Costs will be estimated from two different perspectives, a Portuguese public health care system perspective and a societal perspective. Costs within the public health care system perspective include the cost to run the interventions, including, for instance, therapist costs, as well as other health care costs (other health care utilisation and medication use). In the analysis from a societal perspective, costs beyond the healthcare sector will additionally be included, for instance, social support, and indirect costs (e.g. productivity loss associated with work absenteeism) captured by the UKCC. Costs will be estimated using publicly available unit costs for healthcare services, market process for medications and lost wages for absence from work. Costs per participant will be calculated by multiplying the frequency of each resource by its unit cost. Unit costs will be sourced from publicly available sources.

Costs and QALYs between the Mind programme and the control groups will be compared. Differences in QALYs and costs between the groups will be analysed using generalised linear models to allow for other distributions and functional forms to fit the cost and QALY data [58]. Incremental cost-effectiveness ratios (ICERs) will be estimated, as the ratio between the difference in costs and the difference in QALYs between the Mind programme and the control groups, for each costing perspective. The ICERs will be expressed as additional costs per additional QALY gained. Non-parametric bootstrapping will be carried out to deal with uncertainty around the incremental cost and QALY estimates, which will be represented on cost-effectiveness planes. A cost-effectiveness plane is a scatterplot of the bootstrapped estimates across four quadrants, where each quadrant has a decision implication. Net monetary benefits at different thresholds of willingness to pay will be calculated to determine the proportion of times the Mind programme yields more net benefit than the controls. These proportions will be plotted to generate cost-effectiveness acceptability curves (CEAC), which show the probability of the intervention being cost-effective at different values a decision-maker is willing to pay for a unit of improvement, in this case, a QALY gained [59, 60]. One-way sensitivity analyses will be carried out to test for the impact of changes in assumptions on the robustness of the results.

Aim 3: Determine the contribution of mediators and moderators in treatment effect.

#### Planned and post hoc examination of potential confounds, mediators and moderator effects

Based upon our primary and secondary analyses above, we expect to observe evidence of variables that confound the causal relationship between the intervention and any associated change in outcome over time. Assuming the presence of multiple influential variables, we will use multivariate path analysis to model theoretically-justifiable pathways. This process is expected to require the specification of mediation and moderation pathways, including sequential and competing pathways. For example, a mediation model will be employed if psychological flexibility, self-compassion and mindfulness scores are associated with baseline and longitudinal QoL scores. If participant age is differentially associated with psychological flexibility, self-compassion, mindfulness and QoL scores, then participant age will be added to the model as a moderator (i.e. moderated-mediation), and so on, as necessary.

#### Covariates (controlling variables)

Where variables are expected to correlate with but not differentially predict outcomes, we will control for the influence of expected covariate variables by including them at the baseline model. The planned covariates consist of sociodemographic and clinical variables, such as age, education level, marital status, type of BC, BC stage, previous chemotherapy treatment, use of psychopharmacological medication, time since diagnosis, recurrence and number of major life events occurring during the previous year.

#### Planned moderation analysis variables

Variables such as awareness of psychological processes, frequency of at-home practice and efficacy expectancy are expected to moderate the efficacy of the intervention upon primary and secondary outcomes. These variables will be included as baseline first-order interactions with the intervention allocation factor and post-intervention outcomes. Interactions will be examined at typical proportions of each factor: the mean and ± 1 standard deviation around the mean. Non-significant interaction effects will be trimmed from the model to preserve model parsimony, though significant main effects will be retained and treated as baseline covariates.

### Methods for additional analyses (e.g. subgroup analyses) {20b}

All analyses are described in Sect. 20a, including subgroup analyses.

### Methods in analysis to handle protocol non-adherence and any statistical methods to handle missing data {20c}

All main analysis will be performed as an intention-to-treat (ITT) analysis to provide unbiased estimates of the intervention efficacy regarding the level of participants’ adherence to the study. In such an approach, the participants will be considered according to the group they were originally assigned to regardless of intervention compliance. Missing data will be reported and when MCAR (Missing Completely at Random) or MAR (Missing at Random) occurs, multiple imputation methods will be used to estimate missing values when appropriate. Missing values will be imputed with well-established methods that reduce bias in estimates such as multiple imputation, full information maximum likelihood and empirical Bayes estimation, or multiple imputations with chained equations (MICE), if a substantial amount of missing data exists.

### Interim analyses {21b}

These are low-risk behavioural intervention studies and they were preceded by a pilot study which provided preliminary safety data [29]. Although unlikely, adverse events and clinically significant deterioration will be monitored, discussed and reported (see Sect. 22), following recommendations [61]. The lead statisticians will look for clinically significant deterioration on any outcome at the midpoint of the trials. Clinically significant deterioration will be determined through RCI analyses from baseline to the immediate post-treatment. If clinically significant deterioration is detected, a standardised procedure will follow (see Sect. 22).

### Plans to give access to the full protocol, participant level-data and statistical code {31c}

The study protocol and any de-identified database or statistical code required to support the protocol will be supplied on request.

## Oversight and monitoring

### Composition of the coordinating centre and trial steering committee {5d}

This research does not comprise a large multi-centre RCT, and therefore it is exempted of having a trial coordinating centre. However, the principal investigator (PI) of the project and co-PI play a critical role in coordinating and implementing both RCTs. They provide expertise in planning, conduct, monitoring, analysis and reporting, and convene weekly with the research team.

The project’s Steering Committee (SC) consists of one psychologist, one oncologist and one patient’s representative. SC will convene biannually (or more frequently if required) to oversee the conduct and progress of the trial, adherence to the protocol and the chronogram, participants’ safety and consideration of any new information.

### Composition of the data monitoring committee, its role and reporting structure {21a}

N/A: Given the existence of designated team members for data management, members who monitor data integrity (PI and co-PI) and a SC that oversees participants’ safety, a data monitoring committee will not be established.

### Adverse event reporting and harms {22}

Although unlikely, given the behavioural nature of the interventions and the preliminary data on safety [29], adverse events and clinically significant deterioration will be monitored, discussed and reported, following recommendations [61].

Adverse events and other unintended effects of the trials will be monitored by the therapists of each intervention group. Possible adverse events are as follows: clinically relevant increases in psychological distress, from heightened emotional reactions when confronting difficult thoughts or memories; relevant physical complaints/discomfort (e.g. headaches, muscle tension) from practising unfamiliar exercises, such as the body scan exercise; interpersonal conflict that is disruptive to the group functioning.

Participants will be encouraged to self-report deterioration since the start of the interventions and lead statisticians will look for clinically significant deterioration on any outcome at the midpoint of the trials (see Sect. 21b).

The occurrence of adverse events or clinically significant deterioration will be reported to the involved Ethics Committees and to the SC (see Sect. 5d), who will determine whether they are likely to be related to the intervention and may give recommendations to the PI for the early termination of the trials. The PI has the ultimate authority over early termination, but she will be guided by Linden and Schermuly-Haupt’s [62] stopping rule: if related adverse events or clinically significant deterioration are observed in 20% (approximate base-rate for adverse events in psychotherapies) of the intervention cohort by the trial midpoint, the study will be terminated.

Participants who experience treatment adverse effect or related clinically significant deterioration will be unblinded, removed from the study and provided with appropriate healthcare. As stated before (see Sect. 30), there are no provisions for any additional post-trial care or to provide compensation to those who suffer harm from trial participation.

All adverse events and clinically significant deterioration will also be reported in the trials’ scientific outputs.

### Frequency and plans for auditing trial conduct {23}

N/A: A formal audit of the trial conduct will not be established. However, the institutional sponsor and the funder might ask/perform a random audit.

### Plans for communicating important protocol amendments to relevant parties {25}

Important protocol modifications will be reported to investigators, participants, ethics committees, funder, trial registries and journals.

### Dissemination plans {31a, 31b}

Trial results will be published in peer-reviewed journals and disseminated in scientific meetings and to the press. No professional writers will be used. Authorship eligibility will be determined by ICMJE’s guidelines [63].

## Discussion

This research comprises two pioneering RCTs aiming to test the superiority of an optimised version of the Mind programme, an integrative ACT and compassion-based intervention tailored for women with BC, in improving psychosocial, biological (i.e. immunological and epigenetic) and economic outcomes, compared to an active (i.e. support group) and/or inactive (i.e. treatment as usual group) control condition.

### Limitations

There are several potential limitations to consider. First, the high risk of participant dropout. Patients may not be available or willing to complete post-treatment or follow-up assessments, despite research team efforts to retain study participation (e.g. regular contact, reminders, gifts). Second, recruiting patients already overwhelmed with BC treatment agenda is challenging. Finally, it is possible that our findings may not be generalised to some groups of women with BC (e.g. patients from remote rural areas or digital naïve patients).

### Strengths

First, the pilot study of the Mind programme supported its methodological implementation, and patient and therapist feedback were used to optimise the intervention. Second, the use of an active control group. Compared to the control group(s), participants allocated to the Mind programme are expected to show a higher degree of improvement in cancer-specific quality of life and depressive and anxiety symptoms. Improvements in these outcomes are expected to lead to a concomitant decrease in miRNAs expression and inflammatory biomarkers, as well as to increased cellular immunity (study 1 only). Also, improvements in primary and secondary outcomes are expected to lead to decreases in healthcare resources consumption and in economic costs associated with BC, in both studies. Third, the analysis of the potential mechanisms of change of the Mind programme is an important aspect of the current research. Changes in cancer-specific QoL in the experimental group are expected to be due to improvements in mindfulness, psychological flexibility and self-compassion, and to be maintained over the follow-up assessment, especially in participants that continue mindfulness and compassion practice (prescribed for between-session practice) post-intervention. It is also possible that each intervention will be distinctively beneficial or particularly efficacious for certain subgroups of patients (e.g. higher in psychological distress severity at baseline, younger). This kind of finding would provide important data for treatment customisation in BC. At last, findings from these RCTs will inform about appropriate methods of supporting women with BC who are undergoing radiotherapy, and potentially have important implications for the integration of psychosocial support to optimise service design, delivery and customisation in this population. Findings will also support future studies that may evaluate the Mind programme or other ACT and/or compassion-based interventions for people with cancer, and potentially highlight the clinical relevance of integrative interventions in oncology.

## Trials status, protocol version {3}

Study 1: Recruitment started in January 2023 and is estimated to be complete by March 2025. The current approved protocol (NCT05642897) is version 4 (date of approval: 2024–01–19).

Study 2: Recruitment started in October 2023 and is estimated to be complete by March 2025. The current approved protocol (NCT06212414) is version 1 (date of approval: 2024–01–15).

## Supplementary Information

Additional file 1. SPIRIT checklist.

Additional file 2. Mind Project Team additional members.

Additional file 3. Model consent form.

Additional file 4. Information given to the participant.

## Data Availability

Databases generated by these studies will be made available upon reasonable request to the PI.
