# Correction: Efficacy and cost-effectiveness of an ACT and compassion-based intervention for women with breast cancer: study protocol of two randomised controlled trials {1}

**DOI:** 10.1186/s13063-025-08730-z

**Published:** 2025-06-30

**Authors:** Inês A. Trindade, Andreia Soares, David Skvarc, Diogo Carreiras, Joana Pereira, Óscar Lourenço, Filipa Sampaio, Bruno de Sousa, Teresa C. Martins, Paula Boaventura, Joana Marta-Simões, Mind Project Team, Helena Moreira

**Affiliations:** 1 Center for Health and Medical Psychology, School of Behavioural, Social and Legal Sciences, University of Örebro, Örebro, Sweden; 2 Center for Research in Neuropsychology and Cognitive and Behavioral Intervention,, Faculty of Psychology and Education Sciences, University of Coimbra, Coimbra, Portugal; 3 School of Psychology, Faculty of Health, Deakin University, Geelong, VIC Australia; 4 Lusófona University, Porto, Portugal; 5 CeBER, Faculty of Economics, University of Coimbra, Coimbra, Portugal; 6 Department of Public Health and Caring Sciences, Uppsala University, Uppsala, Sweden; 7 Laboratory of Molecular Pathology, Portuguese Institute for Oncology at Coimbra Francisco Gentil, Coimbra, Portugal; 8 Centre for Neurosciences and Cell Biology, University of Coimbra, Coimbra, Portugal; 9 IPATIMUP Institute of Molecular Pathology and Immunology of the University of Porto, Porto, Portugal; 10 i3s Institute for Research & Innovation in Health, University of Porto, Porto, Portugal; 11 Present address: Miguel Torga Institute (ISMT), Coimbra, Portugal

**Correction: Trials 26, 5 (2025)**

**https://doi.org/10.1186/s13063-024-08626-4**

Following the publication of the original article [1], we were notified the Table including all additional Mind Project Team members was missing, even though this was originally provided and present during the proofing stage.

The original article has now been corrected.
