# Supplementary material for: Efficacy and cost-effectiveness of an ACT and compassion-based intervention for women with breast cancer: study protocol of two randomised controlled trials {1}
Source: Trials. 2025 Jan 3;26:5. doi: 10.1186/s13063-024-08626-4 (PMC11697741; doi:10.1186/s13063-024-08626-4)
Supplement: Supplementary file 2 — Additional file 2. Mind Project Team additional members. [file 13063_2024_8626_MOESM2_ESM.docx]

Mind Project Team additional members (fellow co-authors)

|  | **Affiliation** |
| --- | --- |
| Ana Galhardo | University of Coimbra, Faculty of Psychology and Education Sciences, Center for Research in Neuropsychology and Cognitive and Behavioral Intervention, Coimbra, Portugal; Miguel Torga Institute (ISMT), Coimbra, Portugal. |
| Ana Pereira | University of Coimbra, Faculty of Psychology and Education Sciences, Center for Research in Neuropsychology and Cognitive and Behavioral Intervention, Coimbra, Portugal. |
| Bruna Veloso | University of Coimbra, Faculty of Psychology and Education Sciences, Center for Research in Neuropsychology and Cognitive and Behavioral Intervention, Coimbra, Portugal; Portucalense Infante D. Henrique University, Portucalense Institute for Human Development (INPP), Porto, Portugal |
| Lara Palmeira | Portucalense Infante D. Henrique University, Portucalense Institute for Human Development (INPP), Porto, Portugal |
| Sérgio A. Carvalho | University of Coimbra, Faculty of Psychology and Education Sciences, Center for Research in Neuropsychology and Cognitive and Behavioral Intervention, Coimbra, Portugal; Lusófona University, HEI-Lab: Digital Human-Environment Interaction Lab, School of Psychology and Life Sciences, Lisbon, Portugal |
| Nuno Ferreira | University of Nicosia, Department of Social Sciences, Nicosia, Cyprus |
| Marcela Matos | University of Coimbra, Faculty of Psychology and Education Sciences, Center for Research in Neuropsychology and Cognitive and Behavioral Intervention, Coimbra, Portugal. |
| Paula Castilho | University of Coimbra, Faculty of Psychology and Education Sciences, Center for Research in Neuropsychology and Cognitive and Behavioral Intervention, Coimbra, Portugal. |
| Ricardo João Teixeira | University of Coimbra, Faculty of Psychology and Education Sciences, Center for Research in Neuropsychology and Cognitive and Behavioral Intervention, Coimbra, Portugal. |
| Marta Viegas | Portuguese Institute for Oncology at Coimbra Francisco Gentil, Laboratory of Molecular Pathology, Coimbra, Portugal |
| Margarida Borrego | Coimbra Hospital and University Centre, Radiotherapy Department, Portugal |
| Tomás Cabral Dinis | Coimbra Hospital and University Centre, Radiotherapy Department, Coimbra, Portugal; University of Coimbra, Faculty of Medicine, Coimbra, Portugal |
| Inês Félix Pinto | Coimbra Hospital and University Centre, Radiotherapy Department, Coimbra, Portugal |
| Leonor Santos Martins | Coimbra Hospital and University Centre, Radiotherapy Department, Coimbra, Portugal; University of Coimbra, Faculty of Medicine, Coimbra, Portugal |
| Nicholas J. Hulbert-Williams | Edge Hill University, Department of Psychology, Lancashire, United Kingdom |
