# Supplementary material for: Efficacy and cost-effectiveness of an ACT and compassion-based intervention for women with breast cancer: study protocol of two randomised controlled trials {1}
Source: Trials. 2025 Jan 3;26:5. doi: 10.1186/s13063-024-08626-4 (PMC11697741; doi:10.1186/s13063-024-08626-4)
Supplement: Supplementary file 3 — Additional file 3. Model consent form. [file 13063_2024_8626_MOESM3_ESM.pdf]

NÃO ASSINE ESTE FORMULÁRIO DE CONSENTIMENTO INFORMADO A MENOS QUE TENHA TIDO A OPORTUNIDADE DE PERGUNTAR E TER RECEBIDO RESPOSTAS SATISFATÓRIAS A TODAS AS SUAS PERGUNTAS.

CONSENTIMENTO INFORMADO

De acordo com a Declaração de Helsínquia da Associação Médica Mundial e suas atualizações:

1. Declaro ter lido este formulário e aceito de forma voluntária participar neste estudo.
2. Fui devidamente informada da natureza, objetivos, riscos, duração provável do estudo, bem como do que é esperado da minha parte.
3. Tive a oportunidade de fazer perguntas sobre o estudo e percebi as respostas e as informações que me foram dadas. A qualquer momento posso fazer mais perguntas ao médico responsável do estudo. Durante o estudo e sempre que quiser, posso receber informação sobre o seu desenvolvimento. O Investigador responsável dará toda a informação importante que surja durante o estudo que possa alterar a minha vontade de continuar a participar.
4. Aceito que utilizem a informação relativa à minha história clínica e os meus tratamentos no estrito respeito do segredo médico e anonimato. Os meus dados serão mantidos estritamente confidenciais. Autorizo a consulta dos meus dados apenas por pessoas designadas pelo promotor e por representantes das autoridades reguladoras.
5. Aceito seguir todas as instruções que me forem dadas durante o estudo. Aceito em colaborar com os investigadores e informá-los imediatamente das alterações do meu estado de saúde e bem-estar e de todos os sintomas inesperados e não usuais que ocorram.
6. Autorizo o uso dos resultados do estudo para fins exclusivamente científicos e, em particular, aceito que esses resultados sejam divulgados às autoridades sanitárias competentes.
7. Aceito que os dados gerados durante o estudo sejam informatizados pelo promotor ou outrem por si designado.

8. Posso exercer o meu direito de retificação e/ou oposição. Tenho conhecimento que sou livre de desistir do estudo a qualquer momento, sem ter de justificar a minha decisão e sem comprometer a qualidade dos meus cuidados médicos. Tenho conhecimento que o médico tem o direito de decidir sobre a minha saída prematura do estudo e que me informará da causa da mesma.
9. Fui informado que o estudo pode ser interrompido por decisão do investigador, do promotor ou das autoridades reguladoras.

☐ Aceito participar na intervenção

☐ Não aceito participar na intervenção

Nome da Participante____

Assinatura: ____________________________________

Data: ______/_____/____

Nome de Testemunha(s)

Representante(s)Legal(is):____

____

____

Assinatura: ______________________________________

Data: ______/_____/____

Assinatura: ______________________________________

Data: ______/_____/____

Confirmo que expliquei ao participante acima mencionado a natureza, os objetivos e os potenciais riscos do estudo acima mencionado.

Nome do Investigador:

____

Assinatura: ______________________________________

Data: ______/_____/____

NÃO ASSINE ESTE FORMULÁRIO DE CONSENTIMENTO INFORMADO A MENOS QUE TENHA TIDO A OPORTUNIDADE DE PERGUNTAR E TER RECEBIDO RESPOSTAS SATISFATÓRIAS A TODAS AS SUAS PERGUNTAS.

CONSENTIMENTO INFORMADO

De acordo com a Declaração de Helsínquia da Associação Médica Mundial e suas atualizações:

1. Declaro ter lido este formulário e aceito de forma voluntária participar neste estudo.
2. Fui devidamente informada da natureza, objetivos, riscos, duração provável do estudo, bem como do que é esperado da minha parte.
3. Tive a oportunidade de fazer perguntas sobre o estudo e percebi as respostas e as informações que me foram dadas. A qualquer momento posso fazer mais perguntas ao médico responsável do estudo. Durante o estudo e sempre que quiser, posso receber informação sobre o seu desenvolvimento. O Investigador responsável dará toda a informação importante que surja durante o estudo que possa alterar a minha vontade de continuar a participar.
4. Aceito que utilizem a informação relativa à minha história clínica e os meus tratamentos no estrito respeito do segredo médico e anonimato. Os meus dados serão mantidos estritamente confidenciais. Autorizo a consulta dos meus dados apenas por pessoas designadas pelo promotor e por representantes das autoridades reguladoras.
5. Aceito seguir todas as instruções que me forem dadas durante o estudo. Aceito em colaborar com os investigadores e informá-los imediatamente das alterações do meu estado de saúde e bem-estar e de todos os sintomas inesperados e não usuais que ocorram.
6. Autorizo o uso dos resultados do estudo para fins exclusivamente científicos e, em particular, aceito que esses resultados sejam divulgados às autoridades sanitárias competentes.
7. Aceito que os dados gerados durante o estudo sejam informatizados pelo promotor ou outrem por si designado.

8. Posso exercer o meu direito de retificação e/ou oposição. Tenho conhecimento que sou livre de desistir do estudo a qualquer momento, sem ter de justificar a minha decisão e sem comprometer a qualidade dos meus cuidados médicos. Tenho conhecimento que o médico tem o direito de decidir sobre a minha saída prematura do estudo e que me informará da causa da mesma.
9. Fui informado que o estudo pode ser interrompido por decisão do investigador, do promotor ou das autoridades reguladoras.

☐ Aceito participar na intervenção

☐ Não aceito participar na intervenção

Nome da Participante____

Assinatura: ____________________________________

Data: ______/_____/____

Nome de Testemunha(s)

Representante(s)Legal(is):____

____

____

Assinatura: ______________________________________

Data: _____/_____/____

Assinatura: ______________________________________

Data: _____/_____/____

Confirmo que expliquei ao participante acima mencionado a natureza, os objetivos e os potenciais riscos do estudo acima mencionado.

Nome do Investigador:

____

Assinatura: ______________________________________

Data: _____/_____/____
