# Supplementary material for: Efficacy and cost-effectiveness of an ACT and compassion-based intervention for women with breast cancer: study protocol of two randomised controlled trials {1}
Source: Trials. 2025 Jan 3;26:5. doi: 10.1186/s13063-024-08626-4 (PMC11697741; doi:10.1186/s13063-024-08626-4)
Supplement: Supplementary file 4 — Additional file 4. Information given to the participant. [file 13063_2024_8626_MOESM4_ESM.pdf]

Projeto Mind

É convidada a participar voluntariamente neste estudo porque se encontra a receber tratamento de radioterapia para carcinoma da mama (estadio entre I a III). Este procedimento é chamado consentimento informado e descreve a finalidade do projeto, os procedimentos, os possíveis benefícios e riscos. A sua participação poderá contribuir para melhorar a sua qualidade de vida e saúde mental.

Receberá uma cópia deste consentimento informado para rever e solicitar aconselhamento de familiares e amigos. O Investigador ou outro membro da equipa irá esclarecer qualquer dúvida que tenha sobre o termo de consentimento e também alguma palavra ou informação que possa não entender.

Depois de compreender o estudo e de não ter qualquer dúvida acerca do mesmo, deverá tomar a decisão de participar ou não. Caso queira participar, ser-lhe-á solicitado que assine e date este formulário. Após a sua assinatura e a do Investigador, ser-lhe-á entregue uma cópia. Caso não queira participar, não haverá qualquer penalização nos cuidados que irá receber.

1. INFORMAÇÃO GERAL E OBJETIVOS DO PROJETO

Este projeto de investigação, com a referência PTDC/PSI-GER/7847/2020 e com o título "Programa Mind para doentes oncológicos: Ensaio controlado randomizado para o teste de custo-efetividade e eficácia na mudança de indicadores psicológicos e biológicos em mulheres com carcinoma da mama", é financiado pela Fundação para a Ciência e a Tecnologia, I.P./MCTES através de fundos nacionais (PIDDAC). Decorre no Centro de Investigação em Neuropsicologia e Intervenção Cognitivo-Comportamental (CINEICC), Faculdade de Psicologia e de Ciências da Educação da Universidade de Coimbra, em colaboração com o Serviço de Radioterapia do Centro Hospitalar e Universitário de Coimbra, o Instituto de Patologia e Imunologia Molecular da Universidade do Porto e o Centro de Neurociências e Biologia Celular. Este estudo foi aprovado pela Comissão de Ética do Centro Hospitalar e Universitário de Coimbra (CHUC) de modo a garantir a proteção dos direitos, segurança e bem-estar de todos os doentes ou outros participantes incluídos e garantir prova pública dessa proteção. Como participante neste estudo beneficiará da vigilância e apoio do seu médico, garantindo assim a sua segurança.

Quais são os objetivos do projeto?

Este projeto visa comparar a eficácia de duas intervenções psicológicas em mulheres com carcinoma da mama. Ambas as intervenções têm eficácia cientificamente demonstrada na atenuação do impacto psicológico e melhoria da qualidade de vida de pessoas com cancro. O nosso objetivo é perceber qual é a intervenção mais eficaz e adequada no contexto do cancro da mama.

Como irá decorrer?

Tal será conseguido através de um ensaio clínico controlado e randomizado. As participantes deste estudo receberão uma das duas intervenções durante o período de 8 semanas (uma sessão por semana), através da plataforma zoom, e serão avaliadas antes da intervenção, após a intervenção e seis meses após terminar a intervenção.

A alocação posterior numa intervenção será aleatória. No final do projeto, será oferecida a intervenção que se revelar mais eficaz às participantes que não tiveram oportunidade de a realizar durante o estudo. Os tratamentos e procedimentos médicos manter-se-ão sempre os habituais, para todas as participantes dos dois grupos.

Quem pode participar?

Terão a oportunidade de participar neste projeto mulheres que apresentem as seguintes características:

• Ser acompanhada no Serviço de Radioterapia do CHUC;

• Idade entre 18 e 70 anos;

• Diagnóstico de carcinoma da mama (estadios I-III);

• Não se encontrar a receber psicoterapia, atualmente;

• Não apresentar, atualmente, perturbação psiquiátrica severa (critério a ser avaliado através de uma entrevista breve);

• Ser capaz de compreender e responder a questionários de autorresposta.

2. PROCEDIMENTOS E CONDUÇÃO DO ESTUDO

2.1. Procedimentos

1ª fase – Explicação dos objetivos da investigação e consentimento informado.

2ª fase – Entrevista inicial para avaliação dos critérios de inclusão acima mencionados.

As participantes que não preencherem os critérios de inclusão serão devidamente informadas das razões da impossibilidade de participação e, caso essas razões se devam à presença de perturbação psiquiátrica, receberão recomendações acerca do melhor tipo de apoio médico/psicológico para o seu caso e o/a seu/sua médico/a assistente será notificado/a.

3ª fase – Primeiro momento de avaliação dos indicadores de saúde mental e de medidas fisiológicas, através do preenchimento de um protocolo composto por instrumentos de autorresposta (duração de cerca de 20 minutos) e da colheita de sangue (para avaliação de indicadores imunológicos e epigenéticos).

4ª fase – Aleatorização das participantes elegíveis pelas condições experimentais – intervenção 1 e intervenção 2. Os tratamentos e procedimentos médicos manter-se-ão sempre os habituais, para todas as participantes dos dois grupos.

5ª fase – As participantes iniciam a respetiva intervenção. As intervenções comportarão 8 sessões semanais, via zoom. As intervenções poderão incluir também práticas meditativas que as participantes deverão realizar em casa ao longo do período da intervenção.

6ª fase – No final das intervenções (ou seja, aproximadamente 2 meses após a primeira avaliação), realizar-se-á o segundo momento de avaliação, com o preenchimento de instrumentos de autorresposta.

7ª fase – Período de *follow-up* correspondente à avaliação ao sexto mês após a 6ª fase do projeto, através do preenchimento do protocolo de instrumentos de autorresposta e de uma colheita de sangue.

2.2. Calendário das visitas/ Duração

A sua participação decorrerá ao longo de aproximadamente 8 meses, conforme acima

explicado.

2.3. Tratamento de dados/ Randomização

As participantes serão distribuídas aleatoriamente pela intervenção 1 e 2.

3. RISCOS E POTENCIAIS INCONVENIENTES PARA O DOENTE

Não existem riscos nem efeitos colaterais associados à participação nesta investigação. Esta investigação não envolve intervenção medicamentosa nem procedimentos invasivos ou estudos radiológicos.

4. POTENCIAIS BENEFÍCIOS

As intervenções psicoterapêuticas constantes desta investigação têm por base estratégias psicoterapêuticas reconhecidas e com benefícios cientificamente comprovados no contexto da melhoria da qualidade de vida e da saúde mental.

Além da intervenção, receberá também um Cartão Dá (Sonae) no valor 25€ como recompensa pela sua participação neste projeto, concluídos todos os momentos de avaliação esperados.

5. NOVAS INFORMAÇÕES

Ser-lhe-á dado conhecimento de qualquer nova informação que possa ser relevante para a sua condição ou que possa influenciar a sua vontade de continuar a participar no estudo.

6. SEGURANÇA

Durante a sua participação estará sob a cobertura do seguro de deslocação contratado para o estudo. Se sofrer alguma lesão física como resultado de um acidente durante uma deslocação para e do hospital, calendarizada pelo projeto, será reembolsada pelas despesas médicas necessárias para as tratar.

7. PARTICIPAÇÃO / ABANDONO VOLUNTÁRIO

É inteiramente livre de aceitar ou recusar participar neste estudo. Pode retirar o seu consentimento em qualquer altura sem qualquer consequência para si, sem precisar de explicar as razões, sem qualquer penalidade ou perda de benefícios e sem comprometer a sua relação com o Investigador que lhe propõe a participação neste estudo. Ser-lhe-á pedido para informar o Investigador se decidir retirar o seu consentimento.

O Investigador do estudo pode decidir terminar a sua participação neste estudo se entender que não é do melhor interesse para a sua saúde continuar nele. A sua participação pode ser também terminada se não estiver a seguir o plano do estudo, por decisão administrativa ou decisão da Comissão de Ética. O Investigador do estudo notificá-lo-á se surgir uma dessas circunstâncias, e falará consigo a respeito da mesma.

8. CONFIDENCIALIDADE

Sem violar as normas de confidencialidade, serão atribuídos a auditores e autoridades reguladoras acesso aos registos médicos para verificação dos procedimentos realizados e informação obtida no estudo, de acordo com as leis e regulamentos aplicáveis. Os seus registos manter-se-ão confidenciais e anonimizados de acordo com os regulamentos e leis aplicáveis. Se os resultados deste estudo forem publicados a sua identidade manter-se-á confidencial.

Ao assinar este Consentimento Informado autoriza este acesso condicionado e restrito.

Pode ainda em qualquer altura exercer o seu direito de acesso à informação. Pode ter também acesso à sua informação médica diretamente ou através do seu médico neste estudo. Tem também o direito de se opor à transmissão de dados que sejam cobertos pela confidencialidade profissional.

Os registos médicos que o identificarem e o formulário de consentimento informado que assinar serão verificados para fins do estudo pelo promotor e/ou por representantes do promotor, e para fins regulamentares pelo promotor e/ou pelos representantes do promotor e agências reguladoras noutros países. A Comissão de Ética responsável pelo estudo pode solicitar o acesso aos seus registos médicos para assegurar-se que o estudo está a ser realizado de acordo com o protocolo. Não pode ser garantida confidencialidade absoluta devido à necessidade de passar a informação a essas partes.

Ao assinar este termo de consentimento informado, permite que as suas informações médicas neste estudo sejam verificadas, processadas e relatadas conforme for necessário para finalidades científicas legítimas.

Confidencialidade e tratamento de dados pessoais

Os dados pessoais das participantes no estudo, incluindo a informação médica ou de saúde recolhida ou criada como parte do estudo, (tais como registos médicos ou resultados de testes), serão utilizados para condução do estudo, designadamente para fins de investigação científica e farmacológica relacionados com o medicamento ou com a patologia em estudo.

Ao dar o seu consentimento à participação no estudo, a informação a si respeitante, designadamente a informação clínica, será utilizada da seguinte forma:

1. O promotor, os investigadores e as outras pessoas envolvidas no estudo recolherão e utilizarão os seus dados pessoais para as finalidades acima descritas.
2. Os dados do estudo, associados às suas iniciais ou a outro código que não o(a) identifica diretamente (e não ao seu nome) serão comunicados pelos investigadores e outras pessoas envolvidas no estudo ao promotor do estudo, que os utilizará para as finalidades acima descritas.
3. Os dados do estudo, associados às suas iniciais ou a outro código que não permita identificá-la diretamente, poderão ser comunicados a autoridades de saúde nacionais e internacionais.
4. A sua identidade não será revelada em quaisquer relatórios ou publicações resultantes deste estudo.
5. Todas as pessoas ou entidades com acesso aos seus dados pessoais estão sujeitas a sigilo profissional.
6. Ao dar o seu consentimento para participar no estudo autoriza o promotor ou empresas de monitorização de estudos/estudos especificamente contratadas para o efeito e seus colaboradores e/ou autoridades de saúde, a aceder aos dados constantes do seu processo clínico, para conferir a informação recolhida e registada pelos investigadores, designadamente para assegurar o rigor dos dados que lhe dizem respeito e para garantir que o estudo se encontra a ser desenvolvido corretamente e que os dados obtidos são fiáveis.
7. Nos termos da lei, tem o direito de, através de um dos médicos envolvidos no estudo/estudo, solicitar o acesso aos dados que lhe digam respeito, bem como de solicitar a retificação dos seus dados de identificação.
8. Tem ainda o direito de retirar este consentimento em qualquer altura através da notificação ao investigador, o que implicará que deixe de participar no estudo/estudo. No entanto, os dados recolhidos ou criados como parte do estudo

até essa altura que não a identifiquem poderão continuar a ser utilizados para o propósito de estudo, nomeadamente para manter a integridade científica do estudo, e a sua informação médica não será removida do arquivo do estudo.

9. Se não der o seu consentimento, assinando este documento, não poderá participar neste estudo. Se o consentimento agora prestado não for retirado e até que o faça, este será válido e manter-se-á em vigor.

9. COMPENSAÇÃO DO PARTICIPANTE

Este estudo é da iniciativa do investigador e, por isso, se solicita a sua participação sem uma compensação financeira para a sua execução, tal como também acontece com os investigadores e o Centro de Estudo. No entanto, de forma a compensá-la pelas despesas de deslocação relativas à participação neste projeto, receberá um cartão Dá (Sonae) no valor de 25€, concluídos todos os momentos de avaliação esperados.

10. COMPENSAÇÃO DO CENTRO DE ESTUDO / INVESTIGADOR

O CINEICC não receberá uma compensação financeira pela realização do estudo. Os investigadores proponentes não receberão uma compensação financeira pelo seu trabalho na realização do estudo.

11. CONTACTOS

Se tiver perguntas relativas aos seus direitos como participante deste estudo, deve contactar:

Presidente da Comissão de Ética do CHUC
Centro Hospitalar e Universitário de Coimbra
Praceta Mota Pinto, 3000 075 Coimbra
Telefone: 239 400 400; e-mail: secetica@chuc.min-saude.pt

Se tiver questões sobre este estudo deve contactar:
Andreia Soares | Joana Simões
CINEICC, Faculdade de Psicologia e de Ciências da Educação Universidade de Coimbra
projeto.mind.uc@gmail.com
